# PGE2 Elevates IL-23 Production in Human Dendritic Cells via a cAMP Dependent Pathway

**DOI:** 10.1155/2015/984690

**Published:** 2015-08-27

**Authors:** Quanxing Shi, Zhao Yin, Bei Zhao, Fei Sun, Haisheng Yu, Xiangyun Yin, Liguo Zhang, Shouli Wang

**Affiliations:** ^1^Department of Cardiology, The 306th Hospital, The Chinese People's Liberation Army, Beijing 100101, China; ^2^CAS Key Laboratory of Infection and Immunity, Institute of Biophysics, Chinese Academy of Sciences, Beijing 100101, China

## Abstract

PGE2 elevates IL-23 production in mouse dendritic cells while inhibits IL-23 production in isolated human monocytes. Whether this differential effect of PGE2 on IL-23 production is cell-type- or species-specific has not been investigated in detail. The present study was designed to investigate the effect of PGE2 on IL-23 production in human DCs and the possible underlying mechanisms. Human monocytes derived dendritic cells (Mo-DCs) were pretreated with or without PGE2. Then the cells were incubated with zymosan. Our results demonstrated that PGE2 promoted zymosan-induced IL-23 production in a concentration dependent manner. In addition, it was found that PGE2 is also able to elevate MyD88-mediated IL-23 p19 promoter activity. More importantly, ELISA data demonstrated that db-cAMP, a cAMP analog, and forskolin, an adenylate cyclase activator, can mimic the effect of PGE2 on zymosan-induced IL-23 production, and rp-cAMP, a protein kinase A (PKA) inhibitor, can block the effect of PGE2. Moreover, PGE2 can increase zymosan-induced expression of the mRNA levels of both p19 and p40 subunits, which was mimicked by db-cAMP and forskolin. Our data suggest that PGE2 elevates the production of IL-23 in human Mo-DCs via a cAMP dependent pathway.

## 1. Introduction

While the role of prostaglandins (PGs) has been investigated for decades, their precise roles in the immune response remain contradictory. PGE2 has been demonstrated to exhibit both proinflammatory and anti-inflammatory properties. It has been demonstrated that PGE2 inhibits T-cell activation, while other studies suggest that it possesses proinflammatory and T-cell stimulating properties [1, 2]. Thus, the role of PGE2 as an immune modulator warrants further study.

Interleukin-23 (IL-23), which is a heterodimer consisting of p19 and p40 subunits, is produced by activated antigen-presenting cells, including dendritic cells (DCs) and macrophages. IL-23, a proinflammatory cytokine, is able to induce IL-17 and IFN-*γ* secretion from activated CD4+ T cells and stimulate the proliferation of memory CD4+ T cells [3, 4]. In addition, IL-23 can induce tumor-mediated cytotoxic T-cell responses [5]. Furthermore, IL-23 plays an important role in the pathogenesis of many autoimmune diseases, such as rheumatoid arthritis [6]. Strategies aiming at decreasing the level of IL-23 have been recognized as a promising method against chronic inflammatory diseases [7].

The role of PGE2 in IL-23 production has drawn much attention from many investigators. Study by Sheibanie et al. suggests that PGE2 induces IL-23 production in mouse bone-marrow-derived DCs [8, 9]. The result was confirmed by other groups [10]. Contradictorily, it was reported that PGE2 inhibits IL-23 by human monocytes [11], indicating that the pro- and anti-inflammatory nature of PGE2 with respect to the governing of IL-23 production may be species- or cell-type-dependent. DCs are the bridge between the innate and adaptive immune responses [12, 13]. While there were extensive reports showing a positive effect of PGE2 on IL-23 production by mouse bone-marrow-derived DCs, the effect of PGE2 on human DCs in this respect has not been investigated in detail. There are only sporadic reports regarding the effect of PGE2 on IL-23 production by human Mo-DCs. However, these results are not always consistent. For instance, it was reported that PGE2 promotes IL-23 production [10, 14], while the study by Poloso et al. demonstrated that PGE2 at low concentrations (1–10 nM) promoted IL-23 production via EP4 receptors, and at higher (>50 nM), but still physiologically relevant concentrations (>50 nM), IL-23 was inhibited by an EP2-mediated mechanism [15]. It suggests that the effect of PGE2 may be dependent on its concentration in the microenvironment.

Therefore, the aims of the present study were to thoroughly investigate whether PGE2 is able to affect IL-23 production in human DCs, if this effect depends on cAMP signaling pathway.

## 2. Materials and Methods

### 2.1. Reagents

The following reagents were used: zymosan (Sigma-Aldrich), PGE2 (Sigma-Aldrich), db-cAMP (Sigma-Aldrich), forskolin (Sigma-Aldrich), DMSO (Sigma-Aldrich), and rp-cAMP (Sigma-Aldrich).

### 2.2. Cell Cultures and Transfection

Peripheral blood mononuclear cells (PBMCs) were from healthy human donors (Blood Donors Center of the 307th Hospital) by Ficoll-Paque density gradient centrifugation. Then the cells were resuspended in RPMI 1640 medium supplemented with 20 mM HEPES, 100 U/mL penicillin and streptomycin, 2 mM L-glutamine, 1% nonessential amino acids, and 10% heat-inactivated fetal bovine serum (FBS) (Gibco, USA) and plated in 10 cm dish for 2 h in a 5% CO_2_ incubator. Then the nonadherent cells were discarded. The purity of CD14+ cells by flow cytometry was consistently over 80%. And the viability of cells, as assessed by 7AAD staining, was consistently over 94%. Mono-DCs were obtained by a 5-day culture of plastic-adherent PBMCs in medium with 50 ng/mL GM-CSF (Peprotech) and 12 ng/mL IL-4 (Peprotech) as previously reported [16]. Fresh complete medium was replaced every 3 days. All the blood from healthy donors was obtained according to the National Institutes of Health approved institutional review board protocols.

The human embryonic kidney cell line HEK293T was incubated at 37°C in a 5% CO_2_ incubator with the DMEM medium containing 10% fetal bovine serum, 2 mM L-glutamine, and 100 U/mL penicillin/streptomycin. For the transfection treatment, HEK293T cells were transfected with indicated plasmids using Lipofectamine 2000 reagents (Invitrogen) according to the manufactures' recommendations. Empty vector was used for equalization of the total amount of DNA in each transfection. Subsequently, luciferase assay was performed.

### 2.3. ELISA

The amounts of IL-23 were determined by an ELISA kit for human IL-23 (Mabtech) in supernatants harvested from Mo-DCs according to the manufactures' instructions. The supernatants were routinely diluted twofold before testing. The detection limit for the IL-23 ELISA is 4 pg/mL.

### 2.4. Real-Time Reverse Transcription-Polymerase Chain Reaction (RT-PCR)

The SYBR Green-based RT-PCR technique was used to measure the expression of p19 and p40. The specific primers for real-time PCR were as follows: for p19, sense 5′-CTC­TGC­TCC­CTG­ATA­GCC­CT-3′ and antisense 5′-TGC­GAA­GGA­TTT­TGA­AGC­GG-3′; for p40, sense 5′-GGA­GAG­TCT­GCC­CAT­TGA­GG-3′ and antisense 5′-TCT­TGG­GTG­GGT­CAG­GTT­TG-3′; and for EF1*α*, sense 5′-ATA­TGG­TTC­CTG­GCA­AGC­CC-3′ and antisense 5′-GTG­GGG­TGG­CAG­GTA­TTA­GG-3′. RT-PCR was performed on a Corbett 65H0 machine (Corbett Research, Sydney, NSW, Australia), and the cycling conditions used were 95°C for 15 s and 60°C for 1 min, for 40 cycles. The expression of target gene was normalized to the housekeeping gene EF1*α* messenger RNA from the same sample.

### 2.5. Luciferase Assay

HEK293T cells were seeded in 24-well plates and were transiently transfected with reporter plasmid encoding the firefly luciferase gene under control of the IL-23 p19 promoter together with expression plasmids for MyD88. A 5 kbp fragment of the human IL-23 gene promoter was amplified from human genomic DNA and ligated into the vector pGL4.20 (Promega). The pR-TK encoding Renilla luciferase was used as an internal transfection control. To ascertain the requirement of MyD88 on the upregulation of IL-23 p19 promoter activity, different doses of MyD88 (0–40 ng) were cotransfected with IL-23 p19 (100 ng); then the IL-23 p19 promoter activity was assayed. PGE2 (0–8 *μ*M) and solvent control (DMSO) were added at the beginning of transfection. 6 h later, the medium was changed, and the fresh media containing PGE2 and DMSO were also added, respectively. 40 h later, the cell lysates were collected for luciferase activity detection using the Dual Luciferase Reporter Assay System (Promega, Madison, WI) according to the manufacturer's instructions. Cotransfection with the Renilla luciferase expression vector, pRL-TK (Promega), was used as an internal control. Luciferase units were divided by their renilla control.

## 3. Statistical Analysis

All samples were treated at least in duplicate, and each experiment was repeated at least three times. Data were presented as means ± SEM. Differences between groups were determined by one-way ANOVA followed by Tukey's* post hoc* testing. A value of *P* < 0.05 was considered to be statistically significant.

## 4. Results

### 4.1. PGE2 Promoted Zymosan-Induced IL-23 Production in Mo-DCs but Inhibited IL-23 Production in Human Monocytes

**Figure 1:**
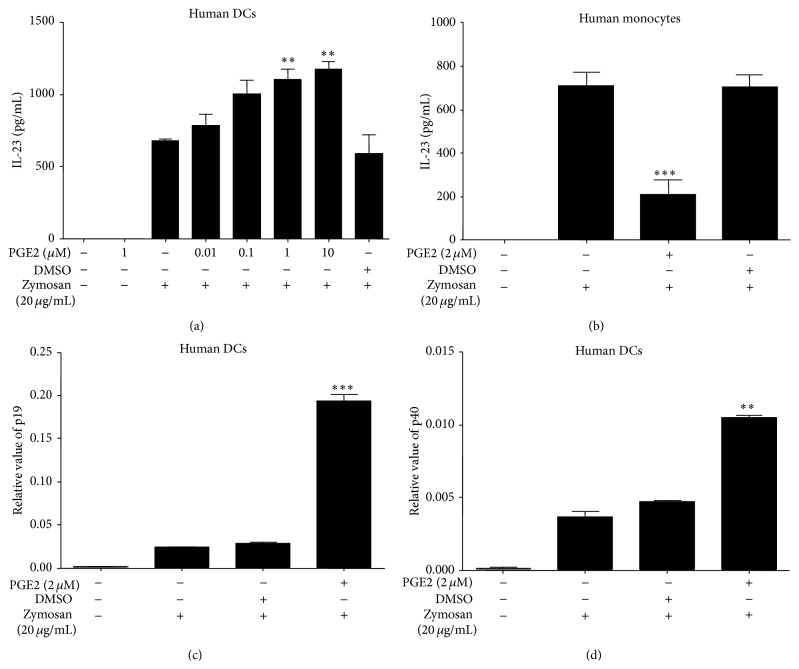
Influence of PGE2 on IL-23 expression in human Mo-DCs and human monocytes. (a, c, and d) Mo-DCs or (b) human monocytes were pretreated for 4 h with PGE2 (0–10 *μ*M), PGE2 (2 *μ*M), or solvent control DMSO. Then the cells were incubated with zymosan (20 *μ*g/mL) in the presence or absence of PGE2 for 24 h (ELISA) or 5 h (RT-PCR). Expression levels of IL-23 protein were determined by ELISA 24 h after stimulation. Expression of (c) IL-23 p19 and (d) IL-23 p40 was analyzed by RT-PCR 5 hours after stimulation. Data represent mean ± SEM of 5 separate experiments. ^*∗∗*^ *P* < 0.01 versus zymosan alone; ^*∗∗∗*^ *P* < 0.001 versus zymosan alone.

To determine the effect of the lipid immunomodulator PGE2 on the ability of human Mo-DCs to produce IL-23 production, Mo-DCs were stimulated with zymosan (20 ug/mL) in the presence or absence of PGE2 for 24 h. It was found that zymosan preferentially induced the production of IL-23 and PGE2 promoted zymosan-induced IL-23 production in a concentration dependent manner (Figure 1(a)). However, it was found that PGE2 significantly inhibited zymosan-induced (20 ug/mL) IL-23 production in human monocytes (Figure 1(b)), which was in accordance with previous reports [11]. Taken together, these results indicated that this differential effect of PGE2 on IL-23 production may be cell-type-specific. PGE2 promoted the IL-23 production at the protein level in human Mo-DCs while inhibiting IL-23 production in human monocytes. IL-23 is a heterodimer consisting of the subunits p19 and p40. We further determined whether PGE2 affected the individual p19 and p40 subunits of IL-23 by RT-PCR. As shown in Figures 1(c) and 1(d), upon zymosan stimulation, there was an upregulation of both p19 and p40 subunits. More importantly, it was observed that PGE2 led to a significant upregulation of p19 and p40 subunits (Figures 1(c) and 1(d)). These results indicated that PGE2 promoted the IL-23 production at both the protein level and RNA level in human Mo-DCs.

### 4.2. PGE2 Elevated MyD88-Mediated IL-23 p19 Promoter Activity

**Figure 2:**
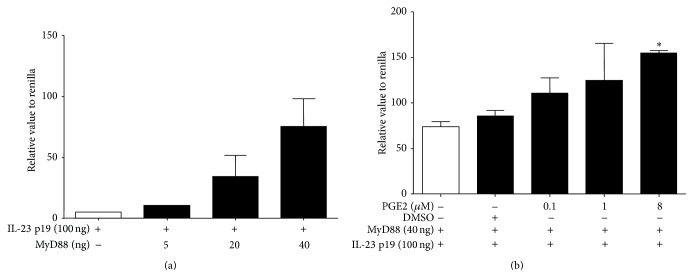
Influence of PGE2 on MyD88-mediated IL-23 p19 promoter activity of HEK293T cells. Different doses of MyD88 (0–40 ng) were cotransfected with IL-23 p19 (100 ng); then the IL-23 p19 promoter activity was assayed (a). In addition, the effect of PGE2 on IL-23 p19 promoter activity was detected (b). In brief, after being pretreated with PGE2 (0.1–8 *μ*M) or DMSO for 1 h, HEK293T cells were transfected with indicated plasmids using Lipofectamine 2000 reagents. Luciferase units were divided by their renilla control, and the empty vector control was then subtracted. Fold change was calculated by dividing each experimental value by the value for the control group. Data are representative of 3 separate experiments. ^*∗*^ *P* < 0.05 versus no treatment.

To investigate the effect of PGE2 on MyD88-mediated IL-23 p19 transcriptional activity, we first ascertained the requirement of MyD88 in the upregulation of IL-23 p19 promoter activity. As shown in Figure 2(a), with the increase of cotransfected MyD88, IL-23 p19 promoter activity significantly increased. More importantly, PGE2 (0–8 *μ*M) acted in a concentration dependent fashion to lead to an upregulation of MyD88-mediated IL-23 p19 transcriptional activity whereas the solvent control DMSO did not possess this ability (Figure 2(b)). The result suggested that PGE2 promotes MyD88-mediated IL-23 p19 promoter activity.

### 4.3. db-cAMP and Forskolin Mimicked the Effect of PGE2 on IL-23 Protein Expression and rp-cAMP Can Partially Block This Effect

**Figure 3:**
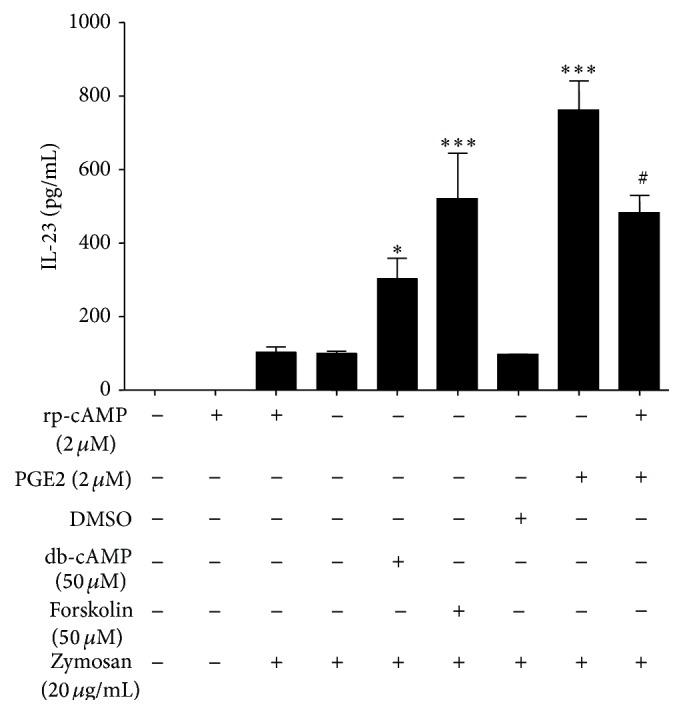
The effect of db-cAMP and forskolin and rp-cAMP on IL-23 expression of zymosan-activated Mo-DCs. After being incubated in the presence or absence of rp-cAMP (2 *μ*M) for 30 min, Mo-DCs were then treated for 4 h with PGE2 (2 *μ*M) or solvent control DMSO or db-cAMP (50 *μ*M) or forskolin (50 *μ*M). Then the cells were incubated with zymosan (20 *μ*g/mL) for 24 h. Expression levels of IL-23 protein were determined by ELISA 24 h after stimulation. Data represent mean ± SEM of 5 separate experiments. ^*∗*^ *P* < 0.05 versus zymosan alone, ^*∗∗∗*^ *P* < 0.001 versus zymosan alone, and ^#^ *P* < 0.05 versus PGE2 plus zymosan treatment.

PGE2 stimulation was demonstrated to be accompanied by the enhancement of cAMP signaling pathway. We aimed to determine whether the promotion effect of PGE2 on IL-23 production by Mo-DCs was cAMP-mediated by applying the cAMP analog db-cAMP, the adenylate cyclase activator forskolin, and the protein kinase A inhibitor rp-cAMP. We quantified IL-23 production from Mo-DCs stimulated with zymosan in the presence or absence of PGE2. As shown in Figure 3, PGE2 was demonstrated to lead to an upregulation of IL-23 as observed in previous experiments (Figure 1(a)). In addition, db-cAMP and forskolin were able to mimic the effect of PGE2, as demonstrated by the fact that the supplementation of the culture medium with db-cAMP and forskolin also promoted IL-23 production in the supernatant of zymosan stimulated Mo-DCs which was similar to that of PGE2 (Figure 3). Moreover, rp-cAMP can partially block the PGE2 effect. The present study strongly indicated that PGE2 promotes IL-23 production in human monocytes via a cAMP dependent pathway.

### 4.4. The Effect of PGE2 on p19 and p40 Subunits of IL-23 Was Mimicked by db-cAMP and Forskolin and rp-cAMP Can Dose Dependently Block This Effect

**Figure 4:**
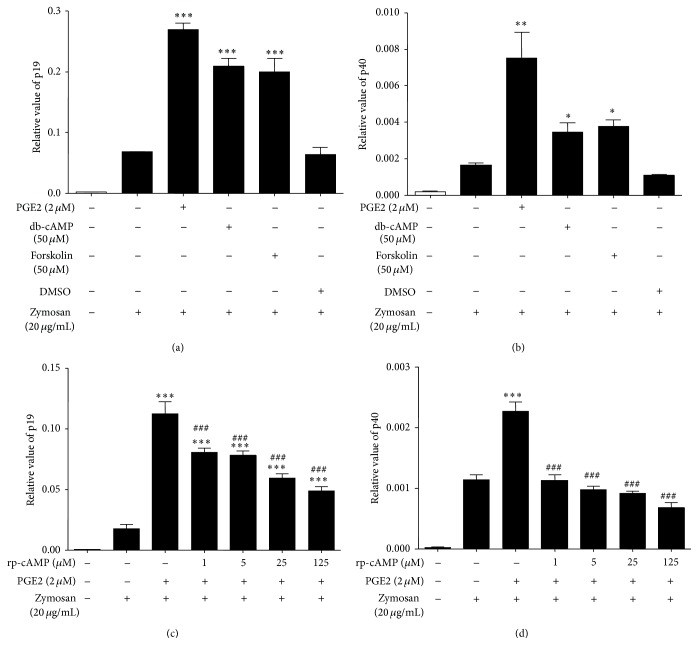
The effect of db-cAMP and forskolin and rp-cAMP on IL-23 RNA expression of zymosan-activated Mo-DCs. Mo-DCs were pretreated for 4 h with PGE2 (2 *μ*M) or solvent control DMSO or db-cAMP (50 *μ*M) or forskolin (50 *μ*M). Then the cells were incubated with zymosan (20 ug/mL) for 5 h. (a) Expression of IL-23 p19 and (b) IL-23 p40 was analyzed by RT-PCR 5 hours after stimulation. After being incubated in the presence or absence of rp-cAMP (1–125 *μ*M) for 30 min, Mo-DCs were then treated for 4 h with PGE2 (2 *μ*M). The cells were incubated with zymosan (20 *μ*g/mL) for 5 h and then (c) expression of IL-23 p19 and (d) IL-23 p40 was analyzed by RT-PCR. Data represent mean ± SEM of 3 separate experiments. ^*∗*^ *P* < 0.05 versus zymosan alone, ^*∗∗*^ *P* < 0.01 versus zymosan alone, ^*∗∗∗*^ *P* < 0.001 versus zymosan alone, and ^###^ *P* < 0.001 versus PGE2 plus zymosan treatment.

To further assess whether the cAMP pathway mediated the upregulation of IL-23, we examined p19 and p40 expression at the RNA levels. As indicated earlier, PGE2 effectively increased IL-23 p19 and p40 production induced by zymosan (Figures 1(c) and 1(d)). The role of cAMP signaling involved in the regulation of IL-23 expression was further demonstrated by the fact that db-cAMP (50 *μ*M) and forskolin (50 *μ*M) were able to mimic the effect of PGE2 (Figures 4(a) and 4(b)). Moreover, rp-cAMP (1–125 *μ*M) can block the PGE2 effect in a concentration dependent manner. This result further demonstrated that the cAMP pathway was involved in the regulation of IL-23 by PGE2.

## 5. Discussion

In the present study, treatment with PGE2 was demonstrated to act in a cAMP dependent manner to elevate IL-23 production in human Mo-DCs. The conclusion is based on the following observations: (1) PGE2 promoted zymosan-induced IL-23 protein production, db-cAMP and forskolin can mimic this effect, and rp-cAMP can partially block the effect of PGE2, (2) PGE2 promoted MyD88-mediated IL-23 p19 promoter activity, and (3) PGE2 promoted the production of p19 and p40 subunits, which was mimicked by db-cAMP and forskolin.

In addition, we have demonstrated that PGE2 promotes zymosan-induced (TLR2 ligand) IL-23 production in human Mo-DCs. These results indicate that PGE2 can synergize TLR2 ligand in the promotion of IL-23 production. Our preliminary experiment showed that neither LPS nor R848 alone was a suitable stimulus for induction of IL-23 production in Mo-DCs (data not shown) while zymosan preferentially induced the production of IL-23. These results suggest that expression of IL-23 by Mo-DCs may possess a complicated mode of activation. As is known to us that zymosan exerts its effect mainly through acting on TLR2 and dectin-1 [16, 17], however, whether the dectin-1 pathway is involved in the induction of IL-23 in the present study warrants further study.

PGE2 has been demonstrated to exhibit both anti- and proinflammatory effect. Nataraj et al. have demonstrated that PGE2 possesses immunosuppressive effects in both T cells and DCs [18]. However, our study strongly suggests that PGE2 is a proinflammatory mediator which promotes the production of IL-23. The differences between the studies may be explained by the differences in model of experiment and cell types. In addition, by using bone-marrow-derived DCs from C57BL/6 mice of different ages, Myer et al. have demonstrated that addition of PGE2 significantly increased IL-23 production by aged DCs whereas IL-23 expression by young DCs was unaffected [19], which indicates that age-related changes in DCs should be revalued. Furthermore, even in the same cell type, there are seemingly contradictory reports that PGE2 differentially regulates Mo-DCs cytokine responses depending on receptor usage [15]. However, in our studies, PGE2 promoted the production of IL-23 in human Mo-DCs at the concentration 0–10 *μ*M. These differences may be explained by the differences in duration of stimuli and mode of action.

However, our study did not provide any additional information about physiologic relevance of the findings* in vivo*. Taking into account the complexity of the regulation of IL-23 production in Mo-DCs, the* in vivo* study in mouse model may need to be explored in future study. In addition, our data only indirectly focused on the mechanism of PGE2 on IL-23 production by using cAMP analogues (db-cAMP), adenylate cyclase activator (forskolin), and protein kinase A (PKA) inhibitor (rp-cAMP), respectively. In addition, forskolin often causes massive increases in cAMP, so it was somewhat disconcerting that forskolin is not as good as PGE2 at enhancing IL-23 (Figures 3 and 4(b)). Because forskolin can have nonadenylate cyclase effects, we cannot preclude the possibility that the nonadenylate cyclase effects of forskolin were involved in the experiment. In addition, we could not preclude the possibility that PGE2 may enhance IL-23 production through cAMP independent pathway. It is of great importance to investigate whether PGE2 as well as forskolin increases cAMP in Mo-DCs in further work.

In conclusion, we have demonstrated that administration of PGE2 elevated zymosan-induced IL-23 production at both RNA and protein levels in human Mo-DCs via a cAMP dependent pathway. The results not only provide new ideas regarding the regulation of IL-23 production in Mo-DCs in theory, but also suggest an additional mechanism for the proinflammatory role of PGE2, particularly important in autoimmune diseases, such as rheumatoid arthritis.
